# Thesis on the Signs of Pregnancy, Presented to the Faculty of the Atlanta Medical College

**Published:** 1856-02

**Authors:** J. Junius Newsome

**Affiliations:** Sandersville, Georgia


### ARTICLE IV.

*Thesis, on the Signs of Pregnancy, presented to the Faculty of the Atlanta Medical College.* By J. JUNIUS NEWSOME, of Sandersville, Georgia, 1855.

Immediately after effective coition of the male with the female, a series of changes ensue, which are of paramount importance to the physician; from the fact that his opinion may decide the fate of the female in a court of justice, where her most important interests are intrusted to the laws of the country.

Aside from a medico-legal point of view, he is often consulted relative to deformities, and diseases which prevent natural labor, or parturition; and should he fail to impart the necessary information, the most disastrous consequences might accrue. His decision may redeem the character of the innocent, and virtuous; restore peace, happiness, and reputation to the family circle; and may cause a fond mother's tears of anguish, to disappear, and brighten up with joy the gloomy face of an aged father, whose agonies would have soon carried his "gray hairs down to the grave with sorrow." Then, let it be the earnest endeavor of every physician, to acquaint himself with rational indications, resulting from fruitful sexual intercourse; for an error committed under the circumstances, might not only affix a lasting stigma upon his own reputation, both professionally and morally, but to that of the chaste and "angelic woman,"

> On whose fair face wild emotions play,
> Lights and shades in sweet confusion blend—
> Shall they depart like closing hours of day,
> And dark disgrace on her soul descend!

Inexperienced, as we are, in the science of obstetrics, we are not expected to present any "signs," save those designated by our illustrious predecessors, whose abilities, in many respects, render them well worthy of example. Yet it is to be ardently regretted, that the "sons of science," in classifying the signs of pregnancy, have fallen into such a discrepancy of opinion; such, however, is the fact; scarcely any two coincide; and this contrariety of opinion, or statement, is no small obstruction in the path of the uninitiated. Therefore, in a consideration of the signs of pregnancy, we shall only allude briefly, to those indications which we conceive to be of most frequent occurrence, and most reliable, consistently with the functions of the organs implicated.

Subsequent to impregnation, there are changes peculiarly characteristic of its existence. The blood is said to be changed in quality and quantity. The fibrin is increased while the action of the pulse is somewhat augmented. Sympathies are excited in remote as well as contiguous organs, and the nervous system may be affected both directly and indirectly; giving rise to that irritability of mind, which so frequently calls into requisition "the woman's weapon of defence." About this period, not unfrequently, the skin undergoes a change in color, especially in women of the lucophlegmatic temperament, in whom it usually becomes sallow *in spots*, whose dimensions vary considerably. We witnessed a case of the kind a year or two since, in which these discolorations were quite perceptible to the most casual observer. These changes are not *usually* considered among the *reputea* or *diagnostic* signs of Pregnancy.

We now proceed to notice some of the special signs commonly particularized by Obstetricians. These we propose considering under two distinct heads, namely: Non-reliable, and Reliable. The former including, 1st, Cessation of Menstruation; 2d, Morning sickness; 3d, Mammary sympaties; 4th, Abdominal enlargement; 5th, Quickening; 6th, Uterine murmur. And the latter, 1st, Ballottement; 2d, Palpitation of the fœtal heart, which is the most reliable sign enumerated.

There are many other signs laid down by Authors, which we consider of minor import, and in fact, only calculated to confuse the junior practitioner in arriving at a definite conclusion. Therefore, we deem their omission beneficial, so far as practicability is concerned; and shall rest satisfied if we can but only do partial justice to the signs previously designated.

*Cessation of Menstruation.*—The non-appearance of the catamenia at the proper period, generally leads the female to suppose that impregnation has occurred, and more especially when she has enjoyed periodical recursions.

Perchance this might be estimated as one of the most unvarying, as it is one of the first, results of conception. Emphatically, however, the cessation of the catamenial discharge is not decisive, for numerous cases are recorded, by high authority, where the menses recurred at the regular intervals during the period of utero-gestation; and indeed, it is said by eminent physiologists, that it requires the unnatural stimulus of impregnation to induce the menstrual discharge in that class of women called viragoes. "If thus," says Dr. Churchill, "menstruation may be suppressed on the one hand, and continued on the other, notwithstanding Pregnancy, it is no proof of conception." But when taken in connection or combination with other signs, its validity is scarcely surpassed by any other in the category.

Time and space will not allow us to advert to the diseases and causes likely to produce cessation of the menses. Therefore, we hasten on to a consideration of the *second* indication under our division of non-reliable, though probable, symptoms.

"*Morning Sickness.*"—The nausea and vomiting attendant about the fifth or sixth week, is confirmatory of the nervous sympathy existing between the uterus and stomach, and in connection with *cessation of menstruation*, is generally considered proof of conception. But when existing alone, "it is certainly a non-reliable sign." The attacks of nausea and vomiting are said to occur soon after rising from a night's repose, and cease within about ten or twenty minutes; and it is from this fact alone that obstetricians and women denominate it the "morning sickness." Almost innumerable instances are on record, where the nausea and vomiting endured for a much longer period than stated above, and indeed the vomiting may become so excessive as to jeopardize the life of the patient.—About the expiration of the third month after conception, it gradually disappears, though it has in some cases, accompanied the entire period of gestation. Authors have had considerable contest as to its value in diagnosticating the existence of Pregnancy. Being yet a mere novice in the great science of Physiology, we cannot advance a theory explanatory of all the various changes incident to impregnation. However, we are quite confident that disease and its concomitants, may be effectual in producing a train of symptoms analogous to those previously alluded to, and in cases where it is utterly impossible for even copulation to have taken place. Dr. Ramsbotham seems to place a great estimate on vomiting, in forming a diagnosis. He remarks, "when vomiting is entirely absent, utero-gestation does not proceed with its usual regularity and activity." Dr. Churchill endorses his statement, and is of opinion that "deviations in other signs" will occur when vomiting is absent. Huston, on the contrary, denies these statements, and says: "I have known women who proceeded regularly through their Pregnancy without experiencing the least degree of sickness." Here we have high authority in direct opposition, and we must pause to determine which is correct—doubtless each one is, according to his observation and experience.

*Mammary Sympathies.*—Here, again, we observe the connection existing between remote organs—the uterus, and the mammary glands. This change in the mamma, engaged the attention of anatomists and physiologists for a series of years; and it was not until after the dissections of the "immortal Lee," that these whimsical speculations were entirely abandoned.

The alteration in these glands are said to be perceptible about the eighth week after conception. The female experiences a variety of peculiar sensations in the organs, which, as a matter of course, are difficult to describe. Authors say they are sensations of palpitation, formication and tingling. We know not the accuracy of such a description; having never consulted a female with regard to these sensations, however, we know there is determination of blood to these organs, producing hardness and distension. The areola, formerly of a pinkish hue, changes its color, and the breast gradually enlarges in proportion to the advancement of pregnancy. The complexion of the individual will modify the color of the areola. Its circle varies in diameter, from three-fourths of an inch to one inch, and deepens in color as the period for delivery approaches. The follicles of Morgagni secrete sufficiently to moisten the female's linen. Milk *may* be formed at quite an early stage, and is generally considered as very good evidence of the existence of pregnancy, but is not conclusive by any means.

The enlargement of the breast, the color of the areola, and the secretion of milk, when taken as symptoms of pregnancy, must be non-reliable; for menstruation in some females, and menostation in others, will produce these identical changes; and to prove the fallacy of the secretion of milk, we have but to refer to Dunglinson's Physiology, where some remarkable cases are cited. Churchill in reference to this subject, says: "No single sign can be relied on in diagnosticating early pregnancy; and it is only when two or three are present, occurring in proper sequence, that we can feel certain." We might continue our comments, but we consider it wholly unnecessary, as the preceding remarks will suffice to convey our opinion on this subject.

*Abdominal Enlargement.*—Conception is followed by a great change in the uterus, and its appendages. There seems to be a demand for nutritious fluid; and to supply it, the uterus takes on a peculiar kind of congestion—its vessels convey a vast quantity of blood, and many which did not carry red blood before, become quite visible. The arteries, veins, nerves, and lymphatics, all participate in the change, and the exterior, or peritoneal coats of the uterus presents a flexiform appearance. The proper tissue of the organ becomes more vascular, and according to Meckle, the parietes thicken from the first to the fourth month. The uterus commences its enlargement at the fundus, after the reception of the ovum, and the enlargement proceeds downwards, until it embraces the cervix. It is retained in the pelvis up to the fourth month; soon after, however, the fundus rises above the symphysis pubis, and gradually ascends until it reaches the ensiform cartilage, which is attained by the expiration of the eighth month. Then it descends somewhat, but still increases in magnitude.

From our foregoing remarks, it would appear that abdominal enlargement is proof positive of conception; yet we must remember the fact, that other causes, not unfrequently, give rise to enlargements of the abdomen. In several instances mentioned by authors, the female herself is of opinion, that she is pregnant, when after due investigation, she is found to be the subject of disease, and not impregnation. The uterus may be enormously distended by an accumulation of air, fluids, or even hydatids; the form of the abdomen in such cases, being nearly, or quite, the same as in pregnancy. Our worthy "Professor of Obstetrics," in his lectures on Disordered Menstruation, related a very interesting case of a married lady, who had imperforate hymen. The catamenia being retained in the uterus, caused its distension, and caused an enlargement of the abdomen, and no doubt provoked symptoms indicative of conception. Therefore, in forming a diagnosis, we must weigh the facts ot the case well. Physicians often confound this stage of pregnancy with abdominal dropsy, and treat it accordingly; when, after an elapse of some months, the cure is spontaneous.

*Quickening.*—This term is applied to the first perception of the movements of the fœtus in utero, on the part of the mother, which most usually occur about the eighteenth week after conception; though it is stated by authors, that these sensations may be experienced much earlier by some than others. Like the preceding signs, it is likely to assume many variations, and capable of causing no small amount of embarrassment to the examiner. The sensation is described as being feeble at first, in most cases; though it may produce "morning sickness," or complete syncope; owing altogether to the temperament, constitution, and general condition of the patient. It gradually acquires force and frequency, until the motion of the different extremities are distinguishable. There seems to be no definite explanation of the movements occurring at the fourth month, and not earlier. Some attribute it to the ascent of the uterus above the symphysis, where it comes in contact with nerves of sensation. This explanation does not seem altogether satisfactory, but as it is more rational than many others advanced, we adopt it, rather as a dernier resort, than a fixed fact. Although this sign is considered by many as conclusive of conception, we maintain that it is *non-reliable*, from the fact, that uterine tumors, flatus, and other foreign substances may not only deceive the patient herself, but the physician likewise, when he is not thoroughly acquainted with these confounding causes, and makes his examination soon after quickening occurs. These views may be directly in conflict with those of our able Professor's. If they are, we trust he will pardon us, as we have not had the opportunity of listening to him on this subject.

We have no doubt that this *sign*, when taken in connection with the preceding, is quite sufficient to form an accurate diagnosis; but when character is at stake, we must not rely on this alone, nor any of the signs of pregnancy, for if we do, our anticipations will often be disappointed. This circumstance leads us to the belief, that we have not given it an incorrect classification among the non-reliable symptoms. I am well aware of the high authority in favor of the validity of this sign, yet, every one will admit, that it is "impaired by the interval which frequently intervenes between the first sensations and their repetition." But we will not pause for contention here, as we are quite confident that our remarks are becoming voluminous.

*Uterine Murmur.*—This appellation has been given to a peculiar intermitting sound heard over the uterine surface. Almost every author makes his own comparison, and so far as we are capable of judging, we think the one made by Dr. Churchill the most appropriate, as it is sufficiently familiar to all. He compares it to the *bruite de souffle* of the heart. It is stated by some writers on this subject, to be limited to the situation of the placenta, while others deny the statement, and declare that it may be heard over any part of the uterus. According to the best authority, the period when it first becomes audible, is about the sixteenth week of utero-gestation. The placenta was formerly supposed to be the seat of these curious sounds. Recent investigators say that it "results from the difference between the calibre of the arteries supplying the uterus, and the uterine sinuses."

We know the blood vessels are greatly increased in size, and consequently carry a greater quantity of blood, and the confluence of an artery and sinus may produce this sound. However, the explanation is a reasonable one at least, and is recognized by high medical authority.

Its value, as a proof of pregnancy, is manifestly doubtful, since the very same sound has been heard to proceed from fibrous tumors in the uterus and ovarian hypertrophy. Therefore, we think our term, *non-reliable*, is admissible.

Having now finished our remarks to the best of our ability, on the first division of the signs of pregnancy, we now proceed to consider the second.

These signs are objected to by some of the fastidious of the profession, for reasons too ridiculous to mention. We contend that they are the paramount signs, and all the indications of the first division combined, are not equal in value to either one of the signs of the latter division.

*Ballottment.*—Having placed the patient in proper attitude, the examiner introduces the index finger into the vagina, and places it upon the cervix uteri, then by gently fillipping the parts above, he will feel a sensation recede, and by permitting the finger to remain motionless, he will, in a few moments, perceive the weight of a body descending on the point of his finger. By operating thus, the fœtus is forced upward into the liquor amnii, and in obedience to the laws ot gravitation, it descends. Some author remarks, that should this body be felt, it is proof positive ot a fœtus in utero; for there is no condition or disease of the organ in which a body can be distinguished floating in the cavity. We feel assured that this is really the case. Doctor Dunglinson observes that, "re-percussion is one of the least equivocal signs of pregnancy; and we might say, that this is the opinion of a majority of authors. Some precautions are necessary before performing ballottment, in order to meet with success. The physician should have his patient in the upright posture, or if she be in the recumbent one, her shoulders should be raised and sustained during the examination.

With regard to the time of practicing repercussion, authors are yet at variance. Some contend that it may be performed as early as the third month, while others say not earlier than the fifth month. Prof. Eve, of the Augusta Medical College, is of opinion that it may be practiced successfully as early as the fourth month. Having no experience on this subject, we are at a loss to say at what time it may be practiced. However, we give preference to the views of Prof. Eve in this respect.

*Pulsation of the Fœtal Heart.*—Unlike the signs preceeding, this one alone is proof positive of Pregnancy; and so far as we know, no author has assumed a position to the contrary. The sounds caused by the systoles and diastoles of the fœtal heart, are peculiar to themselves, and not likely to be mistaken for uterine murmur or any sound emitted by disease. Dr. Churchill thus describes it: "It consists of a rapid succession of short, regular, double pulsations, resembling those of the adult heart except in force and frequency." Kirke and Pagett in considering the frequency of the heart's action, lays down that of the fœtus-in-utero at one hundred and fifty strokes in the minute, but it matters not about the number of pulsations. These, ordinarily, may be declared by the middle of the fourth month. The situation in which the fœtal heart is heard most distinctly, is said to be about the middle or inferior abdominal region, more frequently on the left than the right side.

With this consideration, we close our remarks on the Signs of Pregnancy, not without a feeling of regret that our task has not been better performed. We know there are other signs mentioned by authors, but their minor importance does not demand a consideration in these already prolonged remarks. We hope our interest will call such attention to the profession, we trust to be able soon to enter, as to cause ourself to investigate thoroughly all its various branches. We hope at some future day, to contribute a production that may be of more worth, when experience will help to guide us. Till then, we will leave our pen and this all-important subject, with earnest wishes for the full success of the Institution, which we hope soon to have the honor to represent.

